# Genetic testing and prognosis of sarcomatoid hepatocellular carcinoma patients

**DOI:** 10.3389/fonc.2022.1086908

**Published:** 2023-01-17

**Authors:** Bin Jia, Peiyi Xia, Junqiang Dong, Wenhao Feng, Wenjia Wang, Enjie Liu, Guozhong Jiang, Yanru Qin

**Affiliations:** ^1^ Department of Oncology, The First Affiliated Hospital of Zhengzhou University, Zhengzhou, Henan, China; ^2^ Department of Pathology, The First Affiliated Hospital of Zhengzhou University, Zhengzhou, Henan, China; ^3^ Department of Radiology, The First Affiliated Hospital of Zhengzhou University, Zhengzhou, Henan, China

**Keywords:** genetic testing, prognosis, gene mutation, hepatic tumors, sarcomatoid carcinoma

## Abstract

**Background:**

Sarcomatoid hepatocellular carcinoma (SHC) is a rare epithelial malignancy with high invasiveness and poor prognosis. However, the molecular characteristics and main driver genes for SHC have not been determined. The aim of this study is to explore the potentially actionable mutations of driver genes, which may provide more therapeutic options for SHC.

**Methods:**

In this study, DNA extraction and library preparation were performed using tumor tissues from 28 SHC patients. Then we used Miseq platform (Illumina) to sequence the target-enriched library, and we aligned and processed the sequencing data. The gene groups were tested for SNVs/Indels/CNVs. Tumor mutation burden (TMB) was assessed by the 425-cancer-relevant gene panel. Multivariate analysis of COX’s model was used for survival analysis (OS) of patients’ clinical characteristics.

**Result:**

The median overall survival (OS) of the patients was only 4.4 months. *TP53*, *TERT*, and *KRAS* were the top three frequently mutated genes, with frequencies of 89.3%, 64.3%, and 21.4%, respectively. A considerable number of patients carried mutations in genes involved in the *TP53* pathway (96%) and DNA Damage Repair (DDR) pathway (21%). Multiple potentially actionable mutations, such as *NTRK1* fusions and *BRCA1/2* mutations, were identified in SHCs.

**Conclusions:**

This study shows a landscape of gene mutations in SHC. SHC has high mutation rates in *TP53* pathway and DDR pathway. The potentially actionable mutations of driver genes may provide more therapeutic options for SHC. Survival analysis found that age, smoking, drinking, and tumor diameter may be independent prognostic predictors of SHC.

## 1 Introduction

Sarcomatoid hepatocellular carcinoma (SHC) is a rare epithelial malignancy with high invasiveness and poor prognosis and accounts for no more than 4% of primary malignant hepatic tumors (1, 2). Studies have reported that its occurrence may be related to viral infection, cirrhosis, radiotherapy, chemotherapy, and interventional therapy (3). Typical SHC contains definite malignant epithelial components and spindle cells and other sarcomatoid components in the same tissue, and most scholars believe that the latter is transformed from the former rather than the real mesenchymal tissue (4, 5). Sarcomatoid carcinomas(SC) and carcinosarcomas(CS) are different in pathology and immunohistochemistry. CSalso contain malignant epithelial and sarcomatoid components, but sarcomatoid regions may be composed of different malignant mesenchymal components, such as malignant fibrous histiocytic carcinoma, fibrosarcoma, leiomyosarcoma, osteosarcoma, etc (6). Sarcomatoid areas of SC consist of a single malignant spindle cell and express epithelioid features that can only be detected by immunohistochemistry and electron microscopy. Therefore, different names, including spindle cell carcinoma, pleomorphic carcinoma, and metaplastic carcinoma, can be observed (2).

The pathogenesis of SHC has not been fully clarified yet. The key is the tissue source of sarcomatoid components in the tumor. Histologically, the sarcomatoid cells of SC can exhibit immunoreactivity for cytokeratin, suggesting that SC may originate from the sarcomatoid change of epithelial carcinoma. Many studies suggest a common origin instead of a collision tumor composed of sarcoma and carcinoma (4, 5), however, differential diagnosis between them is still difficult for pathologists. One of the most significant histopathological characteristics that supports SC is the identification of transitional zones between epithelial and mesenchymal cells, but such a “zone” is not always observed (7). Compared with traditional liver cancer, SHC has a high degree of malignancy, fast growth, easy distant metastasis, low surgical resection rate, and recurrence even after radical resection and liver transplantation, eventually, the prognosis is not satisfied (2, 8, 9). The median postoperative survival of SHC patients has been reported as about 8 months, and the 1-year postoperative survival rate of patients is around 22% (1, 8). Due to its rarity, the current literature on these tumors is limited to either case reports or small case series (3, 5, 10).

SHC is insensitive to most chemotherapeutic agents, including conventional platinum-based chemotherapy, leading to an extremely poor prognosis. Meanwhile, little is known about molecular characteristics and main driver genes for SHC, which results in a lack of targeted drugs. In this study, we screened a panel of 425 genes on dissected tumor tissues from 28 SHC patients to show the mutational landscape of SHC, and explore the potentially actionable mutations of driver genes, which may provide more therapeutic options for SHC.

## 2 Materials and methods

### 2.1 Patients

A total of 28 patients with sarcomatoid liver cancer diagnosed by pathology and treated in the First Affiliated Hospital of Zhengzhou University from May 2015 to September 2019 were retrospectively collected in this study. The study was approved by the Review Broad of the First Affiliated Hospital of Zhengzhou University. For each patient, Formalin-fixed paraffin-embedded (FFPE) tumor tissue blocks/sections from biopsies or surgically removed liver lesions were obtained. All specimens of these patients were examined by two experienced pathologists under the guidance of 2019 WHO classification of SHC.

### 2.2 DNA extraction and library construction

DNA extraction, library preparation, and targeted-capture enrichment were performed as previously described (11). Briefly, tissue genomic DNA(gDNA)was extracted from tumor tissue as described using DNeasy Tissue Kits (Qiagen Inc., Germantown, MD, USA). Genomic DNA from the white blood cells(WBCs)was extracted using DNeasy Blood & Tissue Kit (Qiagen Inc., Germantown, MD, USA)and used as the normal control to distinguish germline mutations. The KAPA Hyper Prep kit (Kapa Biosystems, Wilmington, MA, USA) was used for DNA library preparation adapted to an Illumina MiSeq^®^. Customized xGen lockdown probes panel (containing 425 predefined cancer-related genes, Integrated DNA Technologies Inc., Coralville, IA, USA) were used in hybridization enrichment. The capture reaction was performed with Dynabeads M-270 (Life Technologies, Waltham, MA, USA) and the NimbleGen SeqCap EZ Hybridization & Wash Kit (Roche Inc., Indianapolis, IN, USA). Captured libraries were PCR-amplified with KAPA HiFi HotStart ReadyMix (KAPA Biosystems). The purified library was quantified using the KAPA Library Quantification kit (KAPA Biosystems). The target-enriched library was sequenced using Miseq platform (Illumina) with a mean coverage depth of 1000X and 100X for the tumor tissue samples and WBCs, respectively.

### 2.3 Sequence alignment and processing

Sequencing data were demultiplexed by bcl2fastq v2.16.0.10 (Illumina, Inc.), and analyzed by Trimmomatic (12) to remove low-quality (quality<15) or N bases. Then the data were aligned to the hg19 reference human genome with the Burrows-Wheeler Aligner (v0.7.12) (13) with the BWA-MEM algorithm and default parameters to create SAM files. Picard 1.119 (http://picard.sourceforge.net/) was used to convert SAM files to compressed BAM files which were then sorted according to chromosome coordinates. The Genome Analysis Toolkit (14)(GATK, version 3.4-0) was modified and used to locally realign the BAMs files at intervals with indel mismatches and recalibrate base quality scores of reads in BAM files (15).

### 2.4 SNVs/Indels/CNVs/fusions detections

VarScan2 was employed for the detection of single-nucleotide variations (SNVs) and insertion/deletion mutations, followed by annotation using ANNOVAR (16, 17). SNVs were filtered out if the mutant allele frequency (MAF) was less than 0.5% for tumor tissue. Common SNVs were excluded if they were present in >1% of the population in the 1000 Genomes Project or dbSNP database. The resulting mutation list was further filtered by an in-house list of recurrent artifacts based on a normal pool of whole blood samples. Parallel sequencing of matched white blood cells from each patient was performed to further remove sequencing artifacts, germline variants, and clonal hematopoiesis. Copy number variations (CNVs) were detected using ADTEx (http://adtex.sourceforge.net) with default parameters (18). Genomic fusions were identified by FACTERA with default parameters. The fusion reads were further manually reviewed and confifirmed on Integrative Genomics Viewer (IGV).

### 2.5 TMB analysis

Tumor mutation burden (TMB), as assessed by the 425-cancer-relevant gene panel (Nanjing Geneseeq Technology). TMB was defined as the total number of base substitutions and indels in the coding regions of the targeted genes, including synonymous alterations to reduce sampling noise and excluding known driver alterations, as previously described (19).

### 2.6 Statistical analysis

Continuous variables were expressed as mean ± standard deviation (SD) or median (range). The OS rates were estimated by the Kaplan–Meier method, and comparisons were made using the log-rank test. Multivariate analysis was done with the Cox proportional hazard regression model. Statistical analysis was performed using SPSS 21.0 for Windows (SPSS Inc., Chicago, Il, USA). TMB analysis in different gene groups was ranked by Mann–Whitney U test. Overall survival (OS) was calculated from the date of first referral to the date of death (uncensored) or last contact (censored). Multivariate analysis of COX’s model was used for survival analysis (OS) of patients’ clinical characteristics. A p-value ≤0.05 was considered statistically significant.

## 3 Results

### 3.1 Clinical and pathological characteristics of the patient cohort

In the patient cohort, there were 20 males and 8 females, with a male-to-female ratio of 2.5:1. The onset age was 44 ~ 79 years old, and the median age was 55 years old. Eighteen patients were infected with the hepatitis B virus, one with the hepatitis C virus and nine without the hepatitis virus. The tumor site was located in the left lobe of the liver in 7 cases, the right lobe of the liver in 17 cases, multiple livers in 3 cases, and caudate lobe in 1 case. According to the International Union of Cancer (International Union Against Cancer, UICC)/(American Committee on Cancer, AJCC) released the eighth edition of TNM staging of liver Cancer, including 2 stages I cases, 1 stage II case, 9 stage III cases, and 16 stage IV cases. The mean maximum tumor diameter was 6.27cm (1.94cm ~ 14.9cm), as shown in **Table 1**.

**Table 1:** Clinical features of 28 sarcomatoid hepatocellular carcinoma patients.

Clinical features	n	Percentage(%)
Gender		
male	20	71.4
female	8	28.6
Age		
<55	9	32.1
≥50	19	67.9
tumor site		
Left lobe	7	25
Right lobe	17	60.7
Both lobe	3	10.7
Caudate lobe	1	3.6
Tumor size(cm)		
<6	12	42.9
≥6	16	57.1
T stage		
T1	2	7.1
T2	6	21.4
T3	8	28.6
T4	12	42.9
Lymph node metastasis		
NO	14	50
Yes	14	50
Distant metastasis		
NO	23	82.1
Yes	5	17.9
TNM stage		
I	2	7.1
II	1	3.6
III	9	32.1
IV	16	57.2
Therapeutic method		
Surgery	5	17.9
Chemotherapy	4	14.3
Locoregional theraphy^a^	5	17.9
Livertransplantation+adjuvant therapy	1	3.5
Surgery+subsequent therapy^b^	5	17.9
Apatinib	1	3.5
best support care	7	25
hepatitis virus infection		
HBs-Ag(+)	18	64.3
HCV-Ab(+)	1	3.6
No	9	32.1
liver cirrhosis		
Yes	23	82.1
No	5	17.9
AFP(0-20ng/mL)		
<20	25	89.3
≥20	3	10.7
CA-199(0-37ku/L)		
<37	16	57.1
≥37	12	42.9
Ki-67(+)(%)		
<50%	11	39.3
≥50%	17	60.7
ALT(0-40u/L)		
<40	16	57.1
≥40	12	42.9

^a^Local treatments include transcatheter arterial chemoembolization (TACE), radiofrequency ablation (RFA), or a combination of the two.

^b^Follow-up treatments includes postoperative adjuvant chemotherapy, oral targeted drugs, local treatment, and treatment for recurrence or metastasis.

### 3.2 Primary genetic alterations in SHC

A total of 28 SHC tumor samples were analyzed using a 425 gene NGS panel (**Supplemental Table S1**). Overall, 239 genomic alterations of 138 distinct cancer-relevant genes were detected in the SHC tumor samples, including 127 missenses (53%), 24 nonsense (10%), 17 frameshifts (7%), 35 copy number variances (15%), 3 indels (1%), 14 splicing site mutations (6%) and 19 other alterations (8%) (**Supplemental Figure 1**).

**Figure 1:**
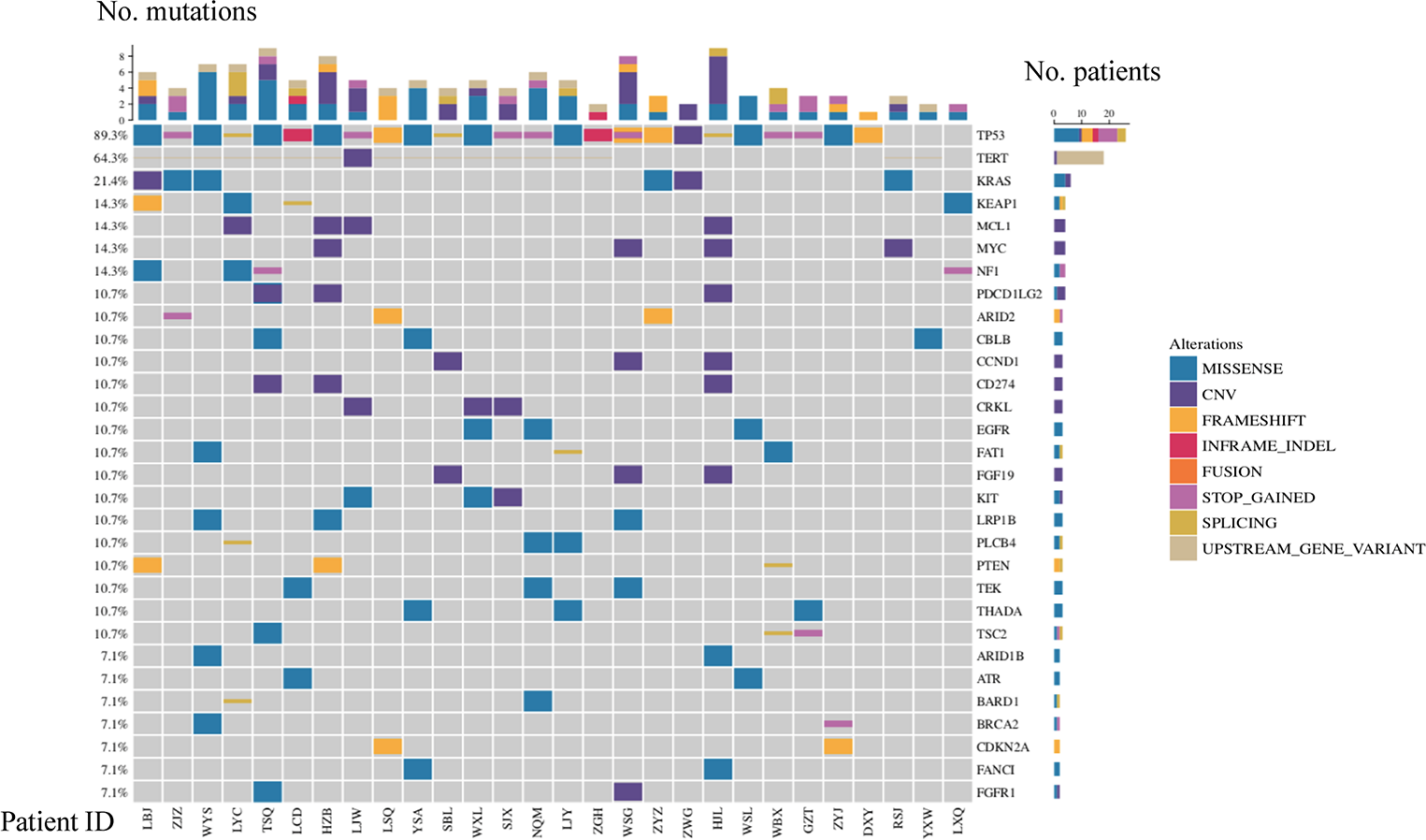
Mutational landscape of SHC.

*TP53*, *TERT*, and *KRAS* were the top three frequently mutated genes, with frequencies of 89.3%, 64.3%, and 21.4%, respectively (**Figure 1**). Alterations of the *TP53* tumor suppressor gene were detected in 25 out of 28 samples in the cohort, indicating that inactivation of the *TP53* pathway was a common genetic event in the SHCs (**Figure 1**). Majority of *TP53* mutated cases were rare mutations and seven nonsense mutations were included, hot spot mutation *R249S* was found in five cases, *R175H* and *R273L* were only found in one case, respectively. Especially, *TP53-ARID1A* co-mutation was found in one case. Seventeen cases showed alterations in *TERT*, among which sixteen cases carried missense mutations in promoter regions, including c.-124C>T in fourteen cases, c.-146C>T, and c.-57A>C in one case, respectively. One case occurred CNV events, which is an amplification of *TERT*. We also found 15 cases with *TERT* mutations harboring *TP53* mutations (15/28, 53.6%). *KRAS* was altered in five patients, including copy number variation (CNV) in one case, G12D in three cases, and G13D in one case.

Other frequently mutated genes included *KEAP1*, *NF1*, *MCL1*, and *MYC* with the same frequency of 14.3% (**Figure 1**). *KEAP1* and *NF1* are tumor suppressor genes, while *MCL1* and *MYC* are oncogenes. The type of mutation in *MCL1* and *MYC* were only copy number variants, and *MCL1* and *MYC* appeared in two cases at the same time. *EGFR* mutations were also detected in three cases (11%, two females, and one male), which were all rare mutations including *P265T*, *A647T*, and *P596L*. Of note, we found a considerable number of patients carrying mutations in genes involved in the *TP53* pathway (96%) and DDR pathway (21%). Based on all gene mutations In patients, *TP53*, *TERT*, *CCND1*, *RB1*, *CDKN2A*, *CTNNB1* and *CDK6* were enriched in the TP53 pathway. It was also found that *ATR*, *POLE*, *BRCA1* and *BRCA2* were enriched in the DDR pathway, respectively (**Supplemental Figure 2**).

Additional actionable driver gene alterations were also identified, including *BRCA1/2* mutations(n=2) and *NTRK1* fusions(n=1). Two cases with *BRCA1/2* pathogenic mutations (*BRCA1* c.5467+1G>A and *BRCA2* p.Q1095*) were likely sensitive to PARP inhibitors (**Figure 1**, **Supplemental Table S2**). Downstream *CRABP2* was jointed to intron 9 of *NTRK1*, and was identified in one patient with *NTRK1* fusion, indicating that treatment of *NTRK1* inhibitors may be a potential therapy.(**Supplemental Table S2**)

### 3.3 TMB distribution in SHC

**Figure 2:**
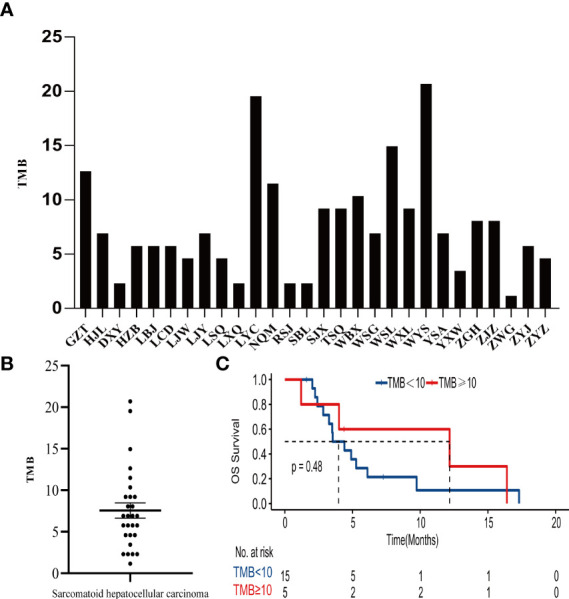
**(A)**Distribution of TMB in each patient, **(B)** the median number of TMB in the SHC patients, **(C)** Kaplan- Meier estimates of overall survival in SHC comparing patients with high TMB and low TMB.

**Figure 3:**
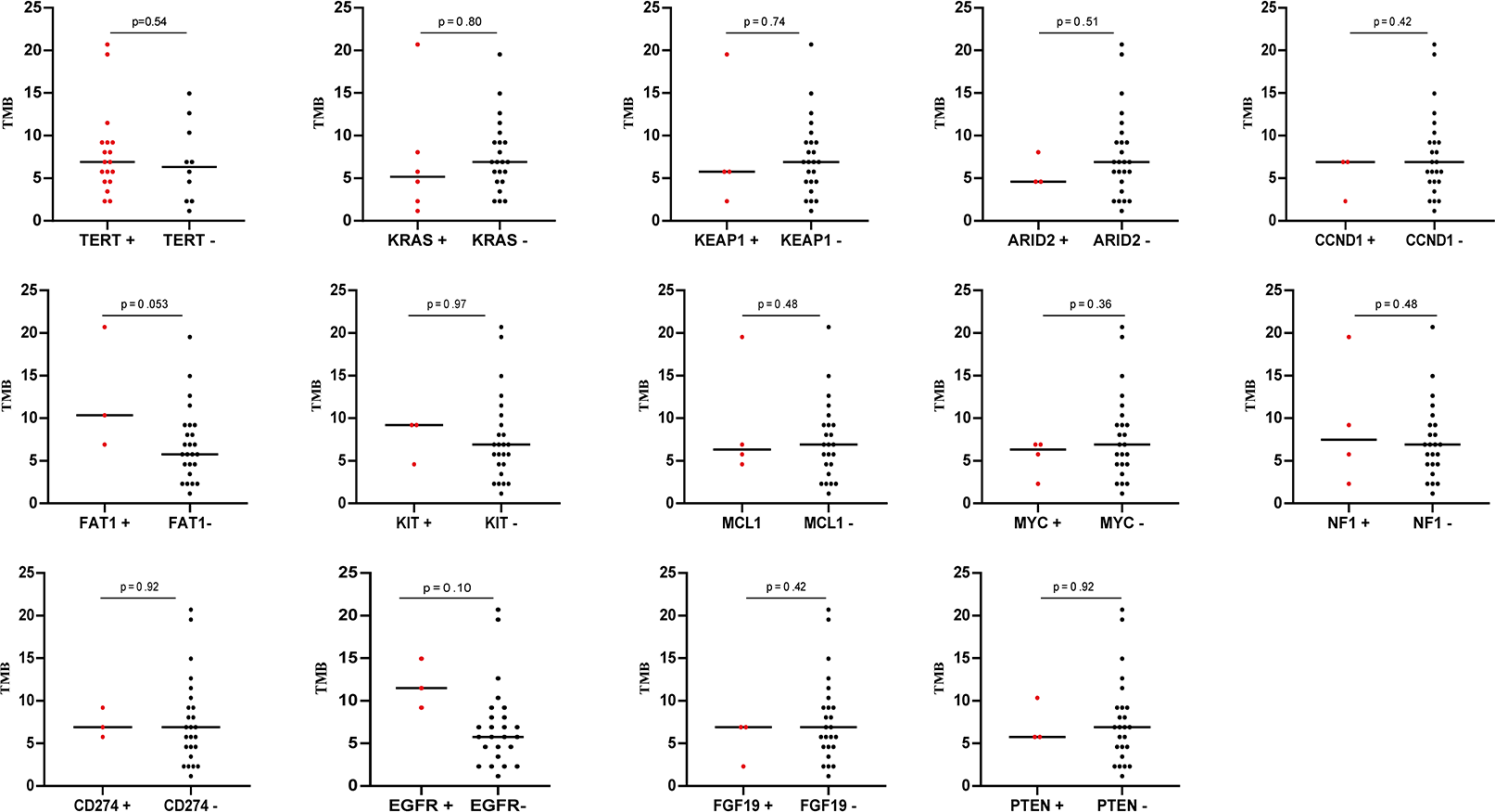
The correlation of TMB with mutation status of different genes.

The tumor mutation burden (TMB) in 28 SHC tumors was ranging from 1.15 to 20.69 mutations/MB with a median number of 6.9 mutations/MB (**Figures 2A, B**, **Supplemental Table S3**). Six (21.4%) patients had high TMB using a clinically validated cut-off of 10 mutations/Mb (20). We also analyzed the association between TMB and OS by stratifying the patients into high TMB (≥10 mutations/Mb) and low TMB (<10 mutations/Mb), but there was no significant difference in OS between these two groups (**Figure 2C**) To further analyze the relationship between genetic alterations and TMB in SHC, the TMB distribution in SHC samples was compared between the wild type gene group and mutated gene group. SHCs with *FAT1* or *EGFR* mutations showed a trend toward higher TMB, but there was no significant difference between the wild-type and mutated groups (**Figure 3**).

### 3.4 Prognostic predictors of SHC

**Figure 4:**
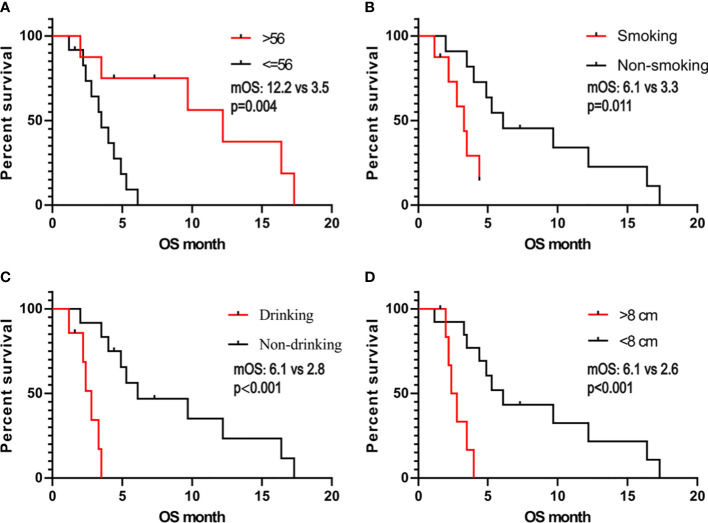
Kaplan- Meier estimates of overall survival in SHC comparing patients with **(A)** age, **(B)** smoking, **(C)** alcohol consumption, and **(D)** tumor diameter.

**Figure 5:**
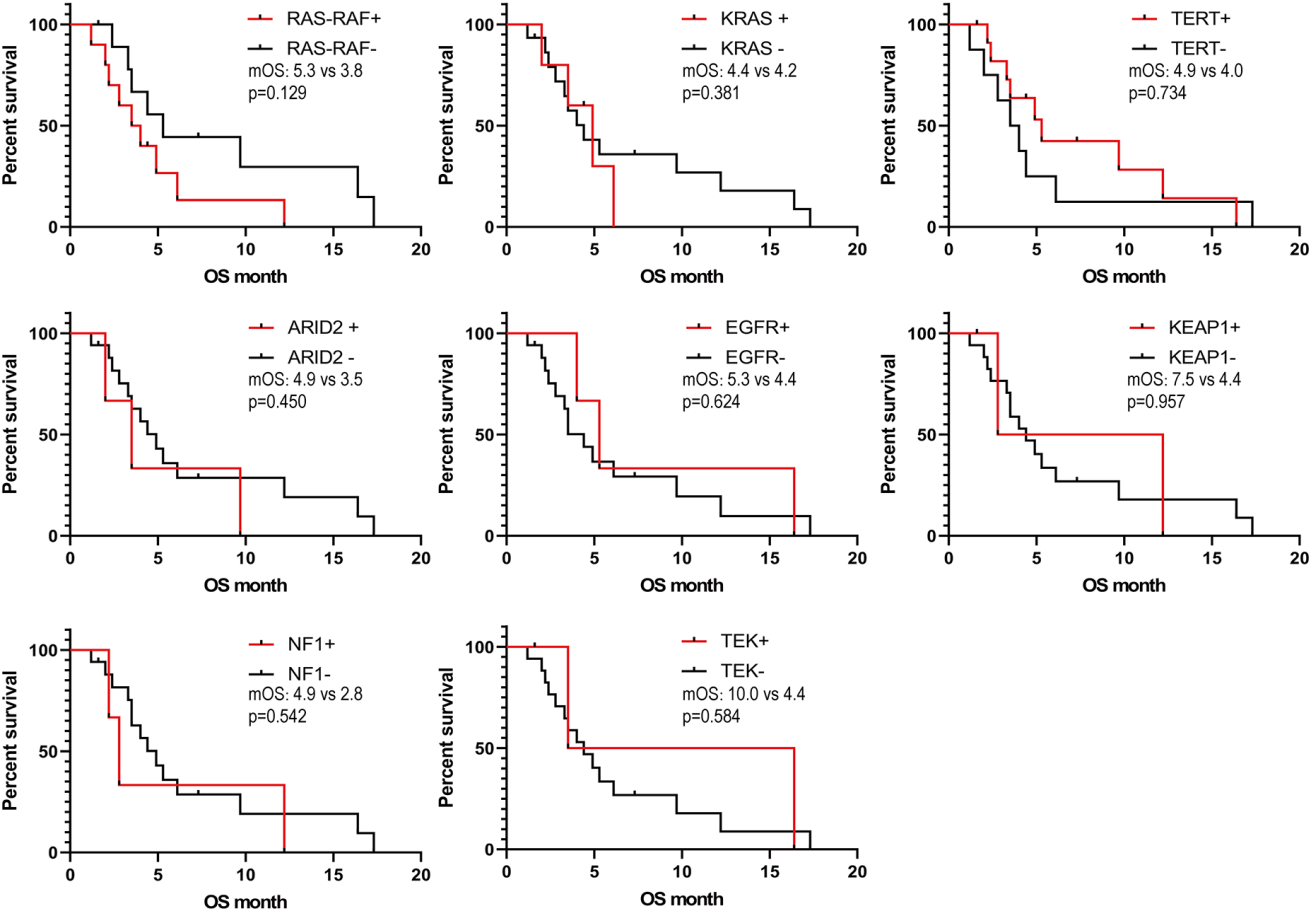
Kaplan- Meier estimates of overall survival in SHC comparing patients with different gene mutations.

We obtained the survival data of 20 patients during the follow-up, 17 patients died and 3 survived by the end of follow-up. The median overall survival (OS) of the patients was only 4.4 months (95%CI: 2.5-6.3 months). The 6-month survival rate was 30.0%, and the 1-year survival rate was 15.0%. Furthermore, we analyzed the correlation of patients’ clinical characteristics with OS using a multivariate COX regression model and found that the clinical variables, including age, smoking, drinking, and tumor diameter, may be independent prognostic predictors of SHC (**Table 2**). Age ≤56 years old was associated with poor prognostic(mOS=12.2 vs 3.5 months, HR 0.18, 95% CI 0.06 to 0.57; log-rank p=0.004; **Figure 4A**), drinking(mOS=2.8 vs 6.1 months, HR 26.54, 95% CI 4.72 to 149.3; log-rank p<0.001), smoking(mOS=3.3 vs 6.1 months, HR 6.81, 95% CI 1.56 to 29.7; log-rank p=0.011), tumor diameter >8 centimeters (mOS=2.6 vs 6.1 months, HR 16.16, 95% CI 3.17 to 82.37; log-rank p<0.001) were also associated with a poor prognostic(**Figures 4B–D**). Then we analyzed the correlation of patients’ genetic alterations with OS and found that mutations in genes in the *RAS/RAF* pathway or *KRAS*, *TERT*, *ARID2*, *EGFR*, *KEAP1*, *NF1*, and *TEK* showed no significant correlation with the survival of SHC patients (**Figure 5**). It’s worth noting that the group with wild-type *KRAS* had a longer mean survival time than those with *KRAS* mutations (6.24 vs 4.18 months). Three patients (21.4%) in the wild-type *KRAS* lived longer than a year, and all patients (0%) in *the KRAS* mutations group died within a year. We compared the treatment between mutated *KRAS* and wild-type *KRAS* groups, finding no statistical difference (**Supplemental Table S4**).

**Table 2:** Univariate and multivariate cox hazards analysis for OS in patients with SHC.

Variable	univariate analysis		Multivariate analysis	
	HR(95%CI)	*P*	HR(95%CI)	*P*
*Age,years*				
≤56 versus >56	7.44(1.58~35.04)	**0.011**	5.81(1.21~27.88)	**0.028**
*Sex*				
Male versus female	1.72(0.59~4.98)	0.321	1.85(0.60~5.74)	0.286
*Tumor location*				
right lobe of liver versus others	0.36(0.12~1.08)	0.067	0.45(0.14~1.42)	0.173
*Tumor_diameter*				
<8 versus>8	0.09(0.02~0.45)	**0.004**	0.12(0.02~0.62)	**0.012**
*radical surgery*				
yes versus no	1.91(0.66~5.56)	0.233	1.93(0.62~6.02)	0.26
*Hepatic history*				
yes versus no	0.44(0.12~1.67)	0.226	0.43(0.11~1.71)	0.229
*tumor_volume*				
>53m^3^versus<53cm^3^	1.59(0.54~4.63)	0.395	1.37(0.44~4.32)	0.59
*drinking*				
yes versus no	22.56(2.61~194.95)	**0.005**	19.16(2.17~168.85)	**0.008**
*smoking*				
yes versus no	7.68(1.54~38.20)	**0.013**	7.29(1.37~38.67)	**0.02**
*ECOG*				
<2 versus =2	0.4(0.11~1.43)	0.159	0.49(0.10~2.47)	0.386

Boldfaced P-value indicates statistical significance.

## 4 Discussion

In this current study, we described the genetic profiles of SHC using 28 FFPE samples and identified *TP53*, *TERT*, and *KRAS* as the most frequent mutations that give new insight into the understanding of the etiology of SHC. In addition, we also indicated the multiple prognostic factors for the progression of SHC. Genetic alterations are key determinants of tumors’ sensitivity to targeted therapies. For example, mutations of *KRAS* and *NRAS* predict resistance to anti-*EGFR* antibodies in colorectal cancers, and patients with lung adenocarcinoma that harbor *EGFR* mutations or *ALK* rearrangements have shown remarkable efficacy in relevant targeted drug therapy (21, 22). Thus, we focus on the effects of genetic mutations in SHC and possible targeted therapies.

*TP53-TERT* co-mutations (53.6%), are much higher than in previous reports (10). Several studies including small samples revealed that the frequency of *TP53* mutations were highest among all gene detected in SHC (10, 23), consistent with our study. Since hepatocellular carcinoma phenotypes are closely associated with gene mutations, *TP53* and *CTNNB1* mutations could define two mutually exclusive groups of distinct phenotypes (24). SHC is more invasive accompanying increased cell proliferation and epithelial-to-mesenchymal transition, corresponding to the characteristics of the “*TP53* mutation group” (24). *KEAP1* is a key factor controlling the endogenous antioxidant response, functioning as a negative regulator of the transcription factor nuclear factor erythroid-2 like 2 *(NFE2L2/NRF2)* (25). Mutations in *KEAP1* are associated with a worse prognosis in cancer (26). It has been suggested that *NF1* was involved in the activation of the *RAS/MAPK* pathway directly; other studies showed *NF1* mutation played an important role in drug resistance to *BRAF*, *EGFR* inhibitors, and tamoxifen, and was associated with shorter survival (27, 28).

Our study showed that the mutation rate of *KRAS* is the highest among oncogenes, followed by *MCL1* and *MYC*. *KRAS* mutations were also detected in another study including 10 cases of liver sarcomatoid carcinomas (23). While the association of *KRAS* mutations with poor prognosis may not be unique to SHCs (29), the occurrence of *KRAS* mutation may induce sarcomatoid phenotype (30) and indicate poor prognosis in cancers with sarcomatoid component (23). *KRAS* is one of front-line sensors that allow the transmission of transducing signals from the cell surface to the nucleus, and affecting a series of essential cellular processes such as cell differentiation, growth, chemotaxis and apoptosis. *KRAS* mutation causes aberrant activation, which is associated spontaneous tumor development in *KRAS*-driven cancer. Meanwhile, *KRAS* mutation regulates tumor microenvironment *via* secreting molecules in a paracrine manner and inducing various chemokines, cytokines and growth factors, which contributes to the promotion and maintenance of malignancy (31). In our study, we found no statistical significance of Kaplan- Meier curves between groups with *KRAS* mutations and wild-type *KRAS*, and it may be due to the different *KRAS* mutation subtypes compared with other studies. *G12D* (60%) is the dominant mutant subtype of *KRAS* in this study, and we still found that the numbers of patients who survived over one year in wild- type *KRAS* group were greater than those in the *KRAS* mutation (n=3 vs n=0). Evidence exists that *KRAS-* mutant cancers with the *G12C* subtype, instead of the *G12D* subtype, was associated with higher TMB and *PD-L1* positivity rate compared with wild-type *KRAS (* 32). Novel inhibitors targeting *KRAS* (*G12C*), such as AMG510 (sotorasib) and MRTX849 (adagrasib), have displayed promising results in preclinical and clinical trials, due to the covalent inhibition of cysteine and high GTPase activity of *KRAS* (*G12C*) (33). *KRAS* itself apart, to target the *KRAS*-driven malignant phenotypes, such as the metabolic vulnerabilities of *KRAS* mutant cancer mentioned in recent review (31), might represent another effective strategy. *MYC* encodes transcription factors associated with cancer cell-cycle progression, proliferation, and biosynthesis (34). Myeloid leukemia 1 (*MCL-1*) is an antiapoptotic protein of the *BCL-2* family that prevents apoptosis by binding to the pro-apoptotic *BCL-2* proteins (35). A previous study showed *MYC* and *MCL1* conferred resistance to chemotherapy by expanding CSCs *via* mtOXPHOS (36).

In line with our findings, *TERT* mutations have been reported in 53.3% of SHC in a previous study (10). *TERT* is the catalytic subunit of telomerase, which activates telomerase to maintain the integrity of telomerase and enables tumor cells to obtain infinite proliferation (37). *TERT* promoter mutations generate novel transcription factor binding sites, contributing to increased *TERT* expression in cancer cells. The presence of *TERT* mutation was associated with worse prognosis in breast cancer, thyroid carcinoma, and lung adenocarcinoma (37), meanwhile, *TERT* promoter mutations are highly associated with sarcomatoid histology in patients with metastatic pleural mesothelioma (38) and urothelial carcinomas (39). Although vaccines (e.g. GV1001) and oligonucleotide inhibitors (e.g. imetelstat) of telomerase have advanced to early stage clinical trials, neither approach has yet demonstrated clinical efficacy, raising questions over their failure to translate (40). Several studies are attempting to specifically target cancer cells harbouring *TERT* promoter mutations, for example, by suppressing *GABPβ1L*-driven transcription at these *de novo ETS* binding sites (41). It is worth noting that *TERT* mutations had little effect on patients’ survival in our study, which may be associated with small samples and poor prognosis of SHC.

We also found a considerable number of patients carrying mutations in genes involved in the TP53 pathway (96%) and DDR pathway (28%). Alterations in DDR genes are associated with genomic instability and increased somatic TMB, which may enhance immunogenicity through increased tumor-specific neoantigen load (42). *ARID1A*, encoding a subunit of the *SWI/SNF* chromatin-remodeling complex, is the most frequently mutated epigenetic regulator in cancers (43). In the endometrial epithelium, the p53 pathway is activated following *ARID1A* loss, and *ARID1A* normally directly represses p53 pathway genes, so *ARID1A* and *TP53* mutations are typically mutually exclusive (44). However, in our study, co-mutations of *TP53-ARID1A* existed in one patient, which may lead to invasive phenotypes including sarcomatoid component (44).

Combination therapy with immune checkpoint inhibitors (ICIs) and an antivascular endothelial growth factor antibody has shown remarkable effects for advanced HCCs in several clinical trials. Unfortunately, SHC patients have often been excluded from key clinical trials with ICIs (45). Luckily, a case report showed a patient with advanced PSHC achieved a complete response to nivolumab after 2 cycles of treatment and the duration of complete remission was longer than 8 months (46). In a large retrospective study of hepatocellular carcinoma(HCC), comprehensive genomic profiling (CGP) of cancer-related genes was performed on 755 consecutive cases of HCC using NGS, finding that the median TMB for the entire cohort was 4 mutations/Mb, with 95% of cases having a TMB of < 10 mutations/Mb (47). Interestingly, the median of TMB was 6.9 mutations/MB in our study, suggesting that SHC may be more likely to have a higher TMB than conventional HCC. Of note, *KRAS* mutant tumor showed prominently increased TMB and remarkable clinical benefit to PD-1 inhibitors in *TP53* or *KRAS* mutant patients, especially those with co-occurring *TP53/KRAS* mutations in cohorts of lung adenocarcinoma immunotherapeutic patients (48). In addition, we observed mutations in *EGFR* or *FAT1* have significantly higher TMB. Three *EGFR* mutations in our study were rare mutations, in contrast to previous studies that reported lower TMB in *EGFR*-mutant lung cancer compared with *EGFR* wild-type (49), we found TMB in *EGFR*-mutant patients was higher in our study, which might be associated with mutation type and the signature of SHC. *FAT1* is a Drosophila tumor suppressor, which has important functions in regulating the *Wnt* pathway and the *Hippo* pathway (50). Mutations in *FAT1* are associated with a higher TMB and lower multiple lymphocyte infiltration (51), which is consistent with our results. Compared with wild-type, patients with *FAT1* mutations could have higher durable clinical benefits during immunotherapy (20). Consequently, such patients might have chance to benefit from immune checkpoint blockade.

Previous studies have reported that the median OS after surgery is about 8 months and the 1-year survival rate is only 22% (1, 8). In our study, the median OS of patients was only 4 months, and the 1-year survival rate was 10%. The prognosis of patients was worse than reported before, which may be related to the high proportion(89.3%) of stage III~IV patients. Survival analysis found that age, smoking, drinking and tumor diameter, may be independent prognostic predictors of SHC. However, the link between genetic alterations and prognosis was not statistically significant. Fortunately, we described some potentially actionable mutations in SHC, including *NTRK1* fusions(n=1) and *BRCA1/2* mutations(n=2), which may provide more treatment options in SHC. BRCA1 and BRCA2 are key regulators of DNA maintenance through homologous recombination (HR) (52). Additionally, they function in DNA crosslink repair as part of the Fanconi anemia (FA) complex and play important roles in the protection of stalled replication forks, transcription regulation, chromatin modulation, cell cycle regulation, checkpoint enforcement and telomere maintenance (52, 53). Meanwhile, DNA repair defects due to BRCA1/2 mutation instigate immune signaling through the cGAS/STING pathway, and the inflammatory signaling provides both tumor-suppressive as well as tumor-promoting traits (52, 54). Mutations in BRCA1/2 confer high-penetrance susceptibility to breast and ovarian cancers, increasing the risk of developing breast cancer by 49–57% and ovarian cancer by 18–40% (55). Poly-adenosine diphosphate ribose polymerase (PARP) inhibitors (PARPi) are effective against tumors with an impaired ability to repair double-strand DNA breaks, and several FDA-approved PARPi are available for treatment of BRCA1/2 carriers with tumors originating at various sites including breast, ovaries, pancreas and prostate (53, 56). However, PARPis used in the clinic remain vulnerable to acquired drug resistance currently, many ongoing clinical trials will evaluate the combination therapy of PARPi and other treatments in breast cancers (56). NTRK fusions, encoding TRK fusion proteins, are oncogenic drivers of a wide variety of adult and paediatric tumors. NTRK gene fusions occur at a low frequency (<1%) in common solid tumors but tend to be high in rare cancers (such as infantile fibrosarcoma and mammary secretory cancer) (57). Luckily, the solid tumors with NTRK fusion can be treated with targeted therapies, such as larotrectinib and entrectinib, the first two TRK inhibitors approved in the United States (58). Many clinical trials have shown good therapeutic results and safety of TRK inhibitors (59, 60). Thus we should encourage broader screening for these fusions in patients with rare tumors as they may benefit from TRK inhibitors, though NTRK fusions are rare.

There are several limitations to our study. First, we did not detect the gene mutation of non-sarcomatoid hepatocellular carcinoma, therefore we could not obtain more specific gene information on SHC through comparison. Second, the cohort size was small, resulting in limited statistical significance between genetic alterations and prognosis.

In conclusion, our study showed a landscape of gene mutations in SHC. *TP53*, *TERT*, and *KRAS* were the top three most frequently mutated genes. Meanwhile, SHC had high mutation rates in the TP53 pathway and DDR pathway. Multiple potentially actionable mutations, such as *NTRK1* fusions and *BRCA1/2* mutations, might provide additional therapeutic options. More samples for genetic variants analysis are still needed for further investigation.

## Data availability statement

The data presented in the study are deposited in the Genome Sequence Archive (Genomics, Proteomics & Bioinformatics 2021) in National Genomics Data Center (Nucleic Acids Res 2022), China National Center for Bioinformation / Beijing Institute of Genomics, Chinese Academy of Sciences that are publicly accessible at https://ngdc.cncb.ac.cn/gsa-human, accession number: HRA003771.

## Ethics statement

The studies involving human participants were reviewed and approved by The Review Broad of the First Affiliated Hospital of Zhengzhou University. The patients/participants provided their written informed consent to participate in this study.

## Author contributions

YQ and GJ conceptualized this work. YQ and GJ supervised the study. BJ, PX, JD, WF, WW, and EL acquisition of data, and BJ, PX, and JD performed the statistical analysis and interpreted data. BJ, PX, and JD prepared the manuscript. YQ, GJ, BJ, PX, JD, WF, WW, and EL revised the manuscript. All authors approved the protocol. All authors contributed to the article and approved the submitted version.

## References

[1] Liao SH Su TH Jeng YM Liang PC Chen DS Chen CH. Clinical manifestations and outcomes of patients with sarcomatoid hepatocellular carcinoma. Hepatology (2019) 69(1):209–21. doi: 10.1002/hep.30162 30014620

[2] Wu L Tsilimigras DI Farooq A Hyer JM Merath K Paredes AZ. Management and outcomes among patients with sarcomatoid hepatocellular carcinoma: A population-based analysis. Cancer (2019) 125(21):3767–75. doi: 10.1002/cncr.32396 31299092

[3] Koo HR Park MS Kim MJ Lim JS Yu JS Jin H. Radiological and clinical features of sarcomatoid hepatocellular carcinoma in 11 cases. J Comput Assist Tomogr (2008) 32(5):745–9. doi: 10.1097/RCT.0b013e3181591ccd 18830104

[4] Dahm HH. Immunohistochemical evaluation of a sarcomatoid hepatocellular carcinoma with osteoclastlike giant cells. Diagn Pathol (2015) 10:40. doi: 10.1186/s13000-015-0274-4 25928039 PMC4411821

[5] Yoshida N Midorikawa Y Kajiwara T Yoshida N Nakayama H Sugitani M. Hepatocellular carcinoma with sarcomatoid change without anticancer therapies. Case Rep Gastroenterol (2013) 7(1):169–74. doi: 10.1159/000350558 PMC3635691 23626518

[6] Wang QB Cui BK Weng JM Wu QL Qiu JL Lin XJ. Clinicopathological characteristics and outcome of primary sarcomatoid carcinoma and carcinosarcoma of the liver. J Gastrointest Surg (2012) 16(9):1715–26. doi: 10.1007/s11605-012-1946-y 22767081

[7] Kan A Guo RP. The prognosis of subsequent surgical treatment in patients with sarcomatoid carcinoma in the liver: A retrospective study. Int J Surg (2018) 55:145–51. doi: 10.1016/j.ijsu.2018.05.736 29860126

[8] Li Z Wu X Bi X Zhang Y Huang Z Lu H. Clinicopathological features and surgical outcomes of four rare subtypes of primary liver carcinoma. Chin J Cancer Res (2018) 30(3):364–72. doi: 10.21147/j.issn.1000-9604.2018.03.08 PMC6037584 30046230

[9] Lin CC Chen CL. Living donor liver transplantation for hepatocellular carcinoma achieves better outcomes. Hepatobiliary Surg Nutr (2016) 5(5):415–21. doi: 10.21037/hbsn.2016.08.02 PMC5075822 27826556

[10] Zhang C Feng S Tu Z Sun J Rui T Zhang X. Sarcomatoid hepatocellular carcinoma: From clinical features to cancer genome. Cancer Med (2021) 10(18):6227–38. doi: 10.1002/cam4.4162 PMC8446410 34331411

[11] Yang Z Yang N Ou Q Xiang Y Jiang T Wu X. Investigating novel resistance mechanisms to third-generation egfr tyrosine kinase inhibitor osimertinib in non-small cell lung cancer patients. Clin Cancer Res (2018) 24(13):3097–107. doi: 10.1158/1078-0432.Ccr-17-2310 29506987

[12] Bolger AM Lohse M Usadel B. Trimmomatic: A flexible trimmer for illumina sequence data. Bioinformatics (2014) 30(15):2114–20. doi: 10.1093/bioinformatics/btu170 PMC4103590 24695404

[13] Li H Durbin R. Fast and accurate short read alignment with burrows-wheeler transform. Bioinformatics (2009) 25(14):1754–60. doi: 10.1093/bioinformatics/btp324 PMC2705234 19451168

[14] McKenna A Hanna M Banks E Sivachenko A Cibulskis K Kernytsky A. The genome analysis toolkit: A mapreduce framework for analyzing next-generation DNA sequencing data. Genome Res (2010) 20(9):1297–303. doi: 10.1101/gr.107524.110 PMC2928508 20644199

[15] Van der Auwera GA Carneiro MO Hartl C Poplin R Del Angel G Levy-Moonshine A. From fastq data to high confidence variant calls: The genome analysis toolkit best practices pipeline. Curr Protoc Bioinf (2013) 43(1110):11.0.1–.0.33. doi: 10.1002/0471250953.bi1110s43 PMC4243306 25431634

[16] Koboldt DC Zhang Q Larson DE Shen D McLellan MD Lin L. Varscan 2: Somatic mutation and copy number alteration discovery in cancer by exome sequencing. Genome Res (2012) 22(3):568–76. doi: 10.1101/gr.129684.111 PMC3290792 22300766

[17] Wang K Li M Hakonarson H. Annovar: Functional annotation of genetic variants from high-throughput sequencing data. Nucleic Acids Res (2010) 38(16):e164. doi: 10.1093/nar/gkq603 20601685 PMC2938201

[18] Amarasinghe KC Li J Hunter SM Ryland GL Cowin PA Campbell IG. Inferring copy number and genotype in tumour exome data. BMC Genomics (2014) 15(1):732. doi: 10.1186/1471-2164-15-732 25167919 PMC4162913

[19] Chalmers ZR Connelly CF Fabrizio D Gay L Ali SM Ennis R. Analysis of 100,000 human cancer genomes reveals the landscape of tumor mutational burden. Genome Med (2017) 9(1):34. doi: 10.1186/s13073-017-0424-2 28420421 PMC5395719

[20] Fang W Ma Y Yin JC Hong S Zhou H Wang A. Comprehensive genomic profiling identifies novel genetic predictors of response to anti-Pd-(L)1 therapies in non-small cell lung cancer. Clin Cancer Res (2019) 25(16):5015–26. doi: 10.1158/1078-0432.Ccr-19-0585 31085721

[21] Solomon BJ Besse B Bauer TM Felip E Soo RA Camidge DR. Lorlatinib in patients with alk-positive non-Small-Cell lung cancer: Results from a global phase 2 study. Lancet Oncol (2018) 19(12):1654–67. doi: 10.1016/S1470-2045(18)30649-1 30413378

[22] Zhu G Pei L Xia H Tang Q Bi F. Role of oncogenic kras in the prognosis, diagnosis and treatment of colorectal cancer. Mol Cancer (2021) 20(1):143. doi: 10.1186/s12943-021-01441-4 34742312 PMC8571891

[23] Ding Y Shao Y Na C Yin JC Hua H Tao R. Genetic characterisation of sarcomatoid carcinomas reveals multiple novel actionable mutations and identifies kras mutation as a biomarker of poor prognosis. J Med Genet (2022) 59(1):10–7. doi: 10.1136/jmedgenet-2020-107083 33115932

[24] Calderaro J Couchy G Imbeaud S Amaddeo G Letouze E Blanc JF. Histological subtypes of hepatocellular carcinoma are related to gene mutations and molecular tumour classification. J Hepatol (2017) 67(4):727–38. doi: 10.1016/j.jhep.2017.05.014 28532995

[25] Rojo de la Vega M Chapman E Zhang DD. Nrf2 and the hallmarks of cancer. Cancer Cell (2018) 34(1):21–43. doi: 10.1016/j.ccell.2018.03.022 29731393 PMC6039250

[26] Zhu G Ren D Lei X Shi R Zhu S Zhou N. Mutations associated with no durable clinical benefit to immune checkpoint blockade in non-S-Cell lung cancer. Cancers (Basel) (2021) 13(6):1397. doi: 10.3390/cancers13061397 PMC8003499 33808631

[27] Bowman L Tiu R Smyth EN Willard MD Li L Beyrer J. Clinical characteristics, treatments, and concurrent mutations in non-small cell lung cancer patients with Nf1 mutations. Clin Lung Cancer (2021) 22(1):32–41 e1. doi: 10.1016/j.cllc.2020.09.011 33221173

[28] Philpott C Tovell H Frayling IM Cooper DN Upadhyaya M. The Nf1 somatic mutational landscape in sporadic human cancers. Hum Genomics (2017) 11(1):13. doi: 10.1186/s40246-017-0109-3 28637487 PMC5480124

[29] Timar J Kashofer K. Molecular epidemiology and diagnostics of kras mutations in human cancer. Cancer Metastasis Rev (2020) 39(4):1029–38. doi: 10.1007/s10555-020-09915-5 PMC7680318 32725342

[30] Fu Y Cruz-Monserrate Z Helen Lin H Chung Y Ji B Lin SM. Ductal activation of oncogenic kras alone induces sarcomatoid phenotype. Sci Rep (2015) 5:13347. doi: 10.1038/srep13347 26289340 PMC4642517

[31] Liu P Wang Y Li X. Targeting the untargetable kras in cancer therapy. Acta Pharm Sin B (2019) 9(5):871–9. doi: 10.1016/j.apsb.2019.03.002 PMC6804475 31649840

[32] Wang S Li Q Ma P Fang Y Yu Y Jiang N. Kras mutation in rare tumors: A landscape analysis of 3453 Chinese patients. Front Mol Biosci (2022) 9:831382. doi: 10.3389/fmolb.2022.831382 35359599 PMC8962378

[33] Huang L Guo Z Wang F Fu L. Kras mutation: From undruggable to druggable in cancer. Signal Transduct Target Ther (2021) 6(1):386. doi: 10.1038/s41392-021-00780-4 34776511 PMC8591115

[34] Dang CV. Myc on the path to cancer. Cell (2012) 149(1):22–35. doi: 10.1016/j.cell.2012.03.003 22464321 PMC3345192

[35] Wang H Guo M Wei H Chen Y. Targeting mcl-1 in cancer: Current status and perspectives. J Hematol Oncol (2021) 14(1):67. doi: 10.1186/s13045-021-01079-1 33883020 PMC8061042

[36] Lee KM Giltnane JM Balko JM Schwarz LJ Guerrero-Zotano AL Hutchinson KE. Myc and Mcl1 cooperatively promote chemotherapy-resistant breast cancer stem cells *Via* regulation of mitochondrial oxidative phosphorylation. Cell Metab (2017) 26(4):633–47 e7. doi: 10.1016/j.cmet.2017.09.009 28978427 PMC5650077

[37] Dratwa M Wysoczańska B Łacina P Kubik T Bogunia-Kubik K. Tert-regulation and roles in cancer formation. Front Immunol (2020) 11:589929. doi: 10.3389/fimmu.2020.589929 33329574 PMC7717964

[38] Tallet A Nault JC Renier A Hysi I Galateau-Salle F Cazes A. Overexpression and promoter mutation of the tert gene in malignant pleural mesothelioma. Oncogene (2014) 33(28):3748–52. doi: 10.1038/onc.2013.351 23975423

[39] Wang X Lopez-Beltran A Osunkoya AO Wang M Zhang S Davidson DD. Tert promoter mutation status in sarcomatoid urothelial carcinomas of the upper urinary tract. Future Oncol (2017) 13(8):705–14. doi: 10.2217/fon-2016-0414 28052688

[40] Guterres AN Villanueva J. Targeting telomerase for cancer therapy. Oncogene (2020) 39(36):5811–24. doi: 10.1038/s41388-020-01405-w PMC7678952 32733068

[41] Mancini A Xavier-Magalhaes A Woods WS Nguyen KT Amen AM Hayes JL. Disruption of the Beta1l isoform of gabp reverses glioblastoma replicative immortality in a tert promoter mutation-dependent manner. Cancer Cell (2018) 34(3):513–28 e8. doi: 10.1016/j.ccell.2018.08.003 30205050 PMC6135086

[42] Ricciuti B Recondo G Spurr LF Li YY Lamberti G Venkatraman D. Impact of DNA damage response and repair (Ddr) gene mutations on efficacy of pd-(L)1 immune checkpoint inhibition in non-small cell lung cancer. Clin Cancer Res (2020) 26(15):4135–42. doi: 10.1158/1078-0432.CCR-19-3529 32332016

[43] Bitler BG Wu S Park PH Hai Y Aird KM Wang Y. Arid1a-mutated ovarian cancers depend on Hdac6 activity. Nat Cell Biol (2017) 19(8):962–73. doi: 10.1038/ncb3582 PMC5541905 28737768

[44] Reske JJ Wilson MR Holladay J Siwicki RA Skalski H Harkins S. Co-Existing Tp53 and Arid1a mutations promote aggressive endometrial tumorigenesis. PloS Genet (2021) 17(12):e1009986. doi: 10.1371/journal.pgen.1009986 34941867 PMC8741038

[45] Morisue R Kojima M Suzuki T Nakatsura T Ojima H Watanabe R. Sarcomatoid hepatocellular carcinoma is distinct from ordinary hepatocellular carcinoma: Clinicopathologic, transcriptomic and immunologic analyses. Int J Cancer (2021) 149(3):546–60. doi: 10.1002/ijc.33545 33662146

[46] Zhu SG Li HB Yuan ZN Liu W Yang Q Cheng Y. Achievement of complete response to nivolumab in a patient with advanced sarcomatoid hepatocellular carcinoma: A case report. World J Gastrointest Oncol (2020) 12(10):1209–15. doi: 10.4251/wjgo.v12.i10.1209 PMC7579730 33133387

[47] Ang C Klempner SJ Ali SM Madison R Ross JS Severson EA. Prevalence of established and emerging biomarkers of immune checkpoint inhibitor response in advanced hepatocellular carcinoma. Oncotarget (2019) 10(40):4018–25. doi: 10.18632/oncotarget.26998 PMC6592287 31258846

[48] Dong ZY Zhong WZ Zhang XC Su J Xie Z Liu SY. Potential predictive value of Tp53 and kras mutation status for response to pd-1 blockade immunotherapy in lung adenocarcinoma. Clin Cancer Res (2017) 23(12):3012–24. doi: 10.1158/1078-0432.CCR-16-2554 28039262

[49] Offin M Rizvi H Tenet M Ni A Sanchez-Vega F Li BT. Tumor mutation burden and efficacy of egfr-tyrosine kinase inhibitors in patients with egfr-mutant lung cancers. Clin Cancer Res (2019) 25(3):1063–9. doi: 10.1158/1078-0432.CCR-18-1102 PMC6347551 30045933

[50] Morris LG Kaufman AM Gong Y Ramaswami D Walsh LA Turcan Ş. Recurrent somatic mutation of Fat1 in multiple human cancers leads to aberrant wnt activation. Nat Genet (2013) 45(3):253–61. doi: 10.1038/ng.2538 PMC3729040 23354438

[51] Martin D Degese MS Vitale-Cross L Iglesias-Bartolome R Valera JLC Wang Z. Assembly and activation of the hippo signalome by Fat1 tumor suppressor. Nat Commun (2018) 9(1):2372. doi: 10.1038/s41467-018-04590-1 29985391 PMC6037762

[52] van Vugt M Parkes EE. When breaks get hot: Inflammatory signaling in Brca1/2-mutant cancers. Trends Cancer (2022) 8(3):174–89. doi: 10.1016/j.trecan.2021.12.003 35000881

[53] Patel PS Algouneh A Hakem R. Exploiting synthetic lethality to target Brca1/2-deficient tumors: Where we stand. Oncogene (2021) 40(17):3001–14. doi: 10.1038/s41388-021-01744-2 33716297

[54] Groelly FJ Porru M Zimmer J Benainous H De Visser Y Kosova AA. Anti-tumoural activity of the G-quadruplex ligand pyridostatin against Brca1/2-deficient tumours. EMBO Mol Med (2022) 14(3):e14501. doi: 10.15252/emmm.202114501 35107878 PMC8899905

[55] Chen S Parmigiani G. Meta-analysis of Brca1 and Brca2 penetrance. J Clin Oncol (2007) 25(11):1329–33. doi: 10.1200/jco.2006.09.1066 PMC2267287 17416853

[56] Menezes MCS Raheem F Mina L Ernst B Batalini F. Parp inhibitors for breast cancer: Germline Brca1/2 and beyond. Cancers (Basel) (2022) 14(17):4332. doi: 10.3390/cancers14174332 PMC9454726 36077867

[57] Cocco E Scaltriti M Drilon A. Ntrk fusion-positive cancers and trk inhibitor therapy. Nat Rev Clin Oncol (2018) 15(12):731–47. doi: 10.1038/s41571-018-0113-0 PMC6419506 30333516

[58] Rolfo C. Ntrk gene fusions: A rough diamond ready to sparkle. Lancet Oncol (2020) 21(4):472–4. doi: 10.1016/s1470-2045(20)30026-7 32105623

[59] Haratake N Seto T. Ntrk fusion-positive non-Small-Cell lung cancer: The diagnosis and targeted therapy. Clin Lung Cancer (2021) 22(1):1–5. doi: 10.1016/j.cllc.2020.10.013 33272813

[60] Demetri GD De Braud F Drilon A Siena S Patel MR Cho BC. Updated integrated analysis of the efficacy and safety of entrectinib in patients with ntrk fusion-positive solid tumors. Clin Cancer Res (2022) 28(7):1302–12. doi: 10.1158/1078-0432.Ccr-21-3597 PMC9365368 35144967

