# Supplementary material for: Genetic testing and prognosis of sarcomatoid hepatocellular carcinoma patients
Source: Front Oncol. 2023 Jan 17;12:1086908. doi: 10.3389/fonc.2022.1086908 (PMC9891294; doi:10.3389/fonc.2022.1086908)
Supplement: Supplementary file 1 [file DataSheet_1.zip › Fig 1.pdf]

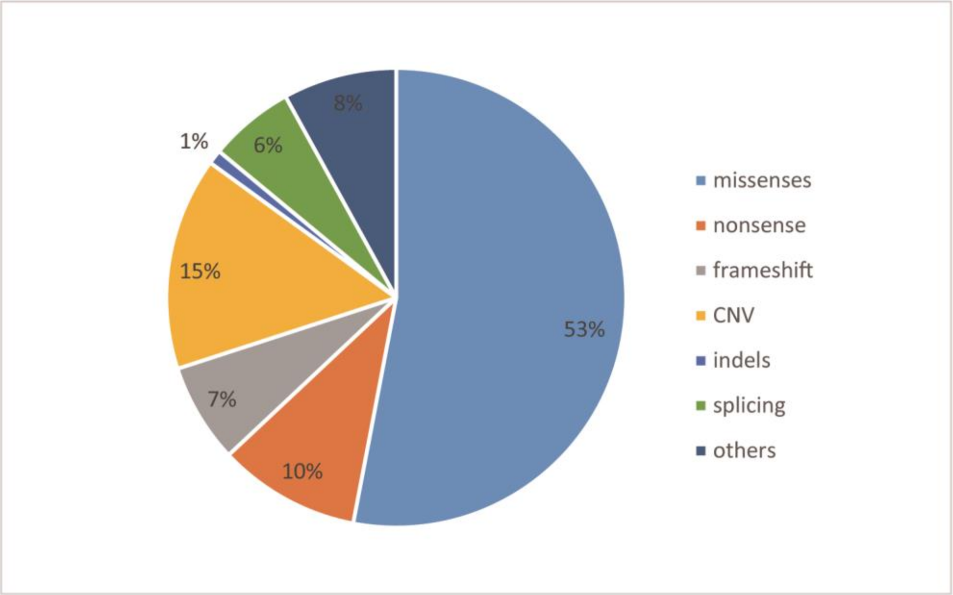

supplement Figure 1: Mutation types of all mutations identified in 28 SHC patients. CNV, copy number variation; Indels, small insertion and deletion; Splicing, splicing site alteration.
