# Supplementary material for: Genetic testing and prognosis of sarcomatoid hepatocellular carcinoma patients
Source: Front Oncol. 2023 Jan 17;12:1086908. doi: 10.3389/fonc.2022.1086908 (PMC9891294; doi:10.3389/fonc.2022.1086908)
Supplement: Supplementary file 1 [file DataSheet_1.zip › Supplemental Table S4.docx]

Supplement Table S4 Treatments between mutated KRAS and wild-type KRAS group

| Therapeutic method | mutated KRAS | wild-type KRAS | *P** |
| --- | --- | --- | --- |
| Surgery | 2 | 1 | 1 |
| Chemotherapy | 0 | 4 |  |
| Locoregional theraphy | 1 | 3 |  |
| Surgery+subsequent [therapy](javascript:;) | 1 | 2 |  |
| best support care | 1 | 5 |  |

*Fisher’s exact test
