# Supplementary figures and images for: Genetic testing and prognosis of sarcomatoid hepatocellular carcinoma patients
Source: Front Oncol. 2023 Jan 17;12:1086908. doi: 10.3389/fonc.2022.1086908 (PMC9891294; doi:10.3389/fonc.2022.1086908)

Supplemental figure.2

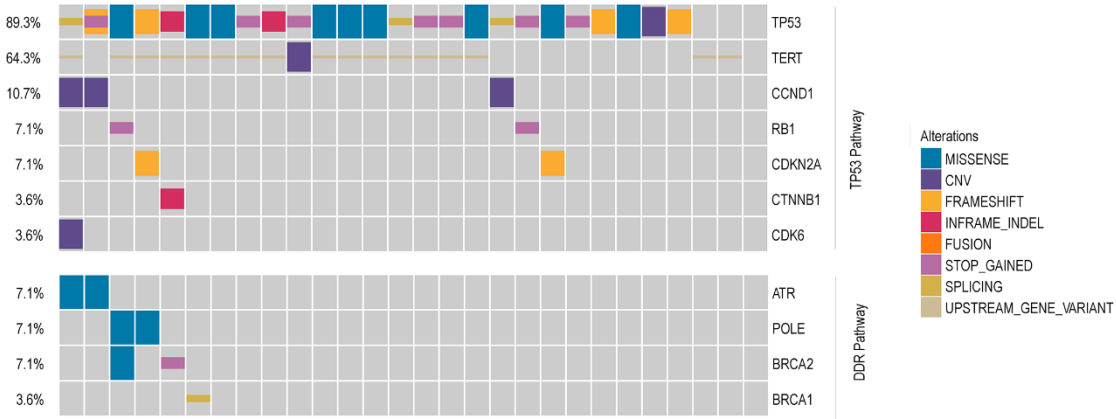

supplement Figure 2: Genes enriched on TP53 pathway and DDR pathway.

Supplement: Supplementary file 1 [file DataSheet_1.zip › Fig 2.pdf]
